# Applications of single-cell transcriptomics in glaucoma research: from the aqueous humor outflow pathway to retinal ganglion cell neurodegeneration

**DOI:** 10.3389/fphys.2026.1953962

**Published:** 2026-09-24

**Authors:** Xiaolu Hao, Dajiang Wang, Zhaohui Li, Shaojun Wang

**Affiliations:** 1 Senior Department of Ophthalmology, Chinese People’s Liberation Army (PLA) General Hospital, Beijing, China; 2 Medical School of the Chinese People’s Liberation Army (PLA) General Hospital, Beijing, China

**Keywords:** aqueous humor outflow pathway, glaucoma, mitochondrial dysfunction, neurodegeneration, neuroinflammation, retinal ganglion cells, single-cell RNA sequencing

## Abstract

Glaucoma is the leading cause of irreversible blindness worldwide, driven by progressive retinal ganglion cell (RGC) degeneration. While genetic and environmental risk factors have been identified, the cell-type-specific mechanisms underlying pathogenesis remain incompletely understood. Single-cell RNA sequencing (scRNA-seq) enables high-resolution analysis of cellular heterogeneity, developmental trajectories and intercellular communication networks. This review synthesizes recent scRNA-seq advances across two critical domains in glaucoma research: (1) aqueous humor outflow pathway (AHOP) physiology and pathology; (2) primary open-angle glaucoma (POAG) pathogenesis. We identify convergent themes, including mitochondrial dysfunction as a shared mechanism across subtypes, the central role of neuroinflammation in RGC degeneration, and the therapeutic potential of metabolic interventions. We further discuss challenges in translating animal models to human disease, integrating multi-species datasets, and resolving cell-type-specific susceptibility. Finally, we highlight emerging directions in spatial transcriptomics, multi-omics integration, and precision medicine.

## Graphical Abstract

**Figure:**
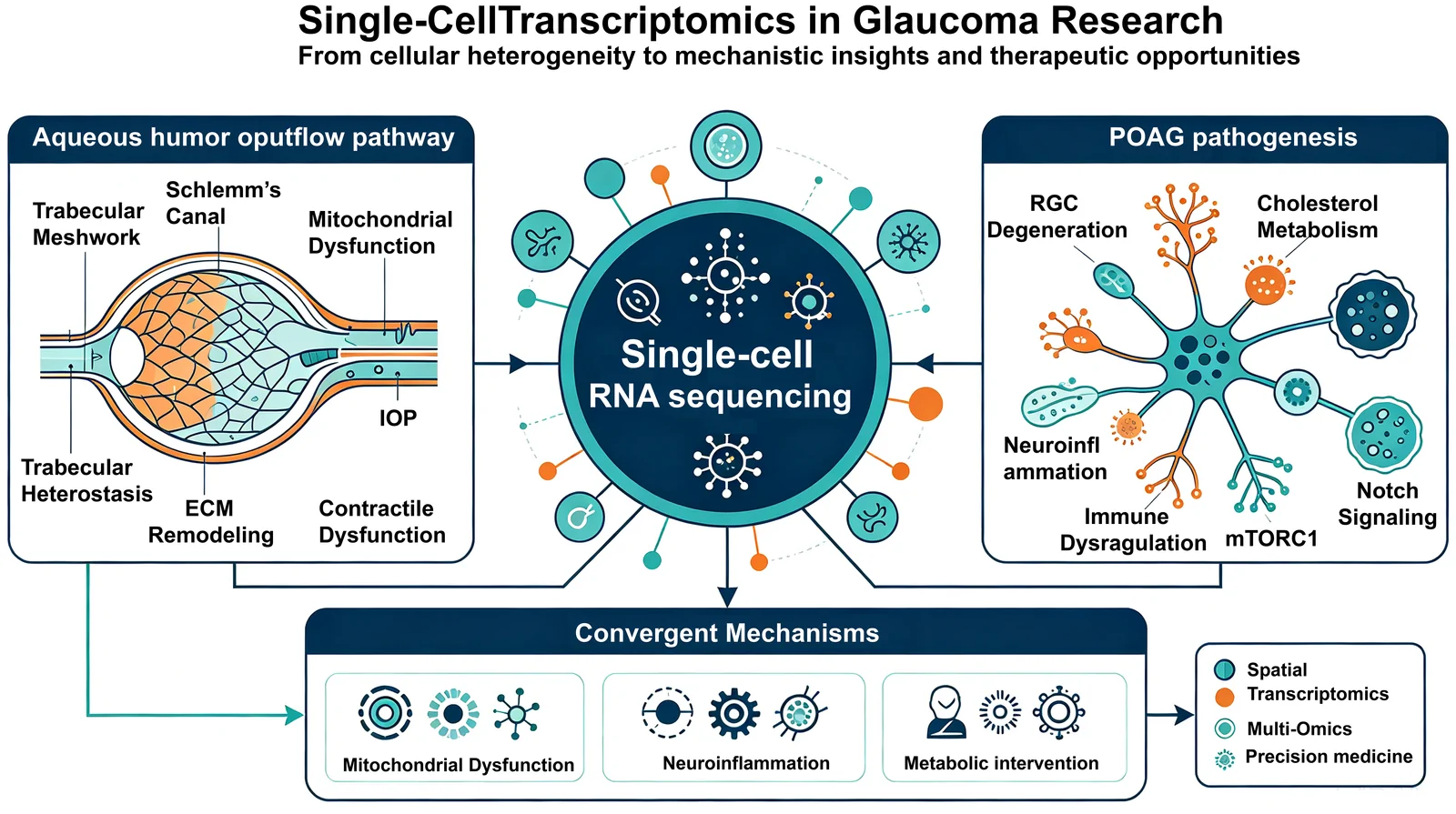
Single cell transcriptomics in glaucoma research: from cellular heterogenity to mechanistic insight and therapeutic opportunities.

## 1 Introduction

### 1.1 Disease burden and clinical challenges

Glaucoma affects approximately 76 million people worldwide, with approximately 10% progressing to bilateral blindness, making it the leading cause of irreversible vision loss (Quigley and Broman, 2006; Global burden and strength of evidence for 88 risk factors in 204 countries and 811 subnational locations, 1990-2021: a systematic analysis for the Global Burden of Disease Study 2021, 2024). The disease encompasses a spectrum of progressive optic neuropathies unified by the characteristic degeneration of RGCs and their axons, resulting in optic disc cupping and corresponding visual field defects. Elevated intraocular pressure (IOP) remains the most important modifiable risk factor; however, the biological basis of glaucomatous neurodegeneration is not fully elucidated. Clinical observations demonstrate that disease progression frequently continues despite adequate IOP control, underscoring the urgent need for non-pressure-dependent neuroprotective strategies.

Glaucoma pathogenesis is multifactorial, involving mechanical stress, ischemia, extracellular matrix (ECM) remodeling, excitotoxicity, oxidative stress, and aberrant immune responses (Weinreb et al., 2014). The retina and optic nerve head contain dozens of distinct cell types, each contributing uniquely to homeostasis and disease. During RGC injury, diverse cell populations exhibit distinct molecular signatures that may drive disease progression. While bulk RNA sequencing has been valuable for identifying broad transcriptional changes, it cannot resolve the molecular features of individual cell types within complex tissues. Moreover, RGCs themselves constitute a highly diverse population, with over 45 molecularly defined subtypes identified in mice, each displaying differential susceptibility to injury (Sanes and Masland, 2015). This cellular complexity necessitates higher-resolution analytical approaches.

### 1.2 Advantages of single-cell transcriptomics

Single-cell RNA sequencing enables transcriptomic analysis at single-cell resolution, revealing cell-type-specific gene expression patterns, reconstructing developmental trajectories through pseudotemporal ordering, and mapping intercellular communication networks via ligand–receptor interaction analysis (Papalexi and Satija, 2018). Since its initial application to retinal tissue, scRNA-seq has been systematically deployed across various ophthalmic diseases, including myopia, corneal disorders, uveitis, retinal degeneration, and ocular tumors (Cheng et al., 2025). In glaucoma research specifically, scRNA-seq has catalyzed paradigm shifts in our understanding of trabecular meshwork biology, RGC subtype susceptibility, neuroinflammatory mechanisms, and therapeutic target identification.

This review provides a comprehensive synthesis of scRNA-seq applications in glaucoma research, organized into two major thematic areas-AHOP and POAG-with subsections covering cross-species atlases, TM-SC signaling, contractile dysfunction, aging and mitochondrial biology, spatial transcriptomics, cholesterol metabolism, genetic integration, systemic immunity, RGC subtype vulnerability, and optic nerve head remodeling. Rather than providing sequential study summaries, we critically evaluate areas of consensus, conflicting findings, technical limitations, and translational relevance. We identify convergent pathogenic mechanisms across different glaucoma models and subtypes, explicitly distinguish association from causation, and discuss current controversies and research gaps limiting clinical translation.

## 2 Single-cell dissection of the aqueous humor outflow pathway

The aqueous humor outflow pathway (AHOP), comprising the trabecular meshwork (TM) and Schlemm’s canal (SC), regulates IOP by controlling aqueous humor drainage resistance. Dysfunction of this pathway represents the primary pathophysiological mechanism in POAG and some other secondary glaucoma (Gabelt and Kaufman, 2005). scRNA-seq has fundamentally advanced our understanding of cellular composition, heterogeneity, and disease-associated alterations in the AHOP across humans and model organisms.

### 2.1 Cross-species single-cell atlases of the AHOP

Patel et al. first established the foundational single-cell atlas of human AHOP by analyzing 8, 758 cells from eight donor eyes, identifying 12 distinct cell types and revealing substantial heterogeneity within TM populations (Patel et al., 2020). A key finding was that Schlemm’s canal (SC) endothelial cells exhibit a unique hybrid vascular/lymphatic gene expression signature, challenging the traditional classification of SC as a purely vascular structure. Concurrently, van Zyl et al. constructed a cross-species comparative atlas encompassing humans, cynomolgus monkeys, rhesus macaques, pigs, and mice based on approximately 24, 000 single-cell transcriptomes. This study identified 19 cell types, validated molecular markers histologically, and revealed both conserved and divergent gene expression patterns across species (van Zyl et al., 2020). Notably, while many human cell types had clear orthologs in animal models, significant differences in cell-type proportions and expression patterns raise important questions about the translational validity of preclinical models. These foundational atlases established that TM and SC are far more heterogeneous than previously appreciated. However, the studies are descriptive rather than mechanistic, and the functional significance of the identified subtypes remains largely unexplored. The species differences observed, particularly between human and mouse, suggest that findings from rodent models require cautious interpretation and validation in human tissues.

### 2.2 TM-SC signaling in congenital and primary glaucoma

Patients with high-pressure glaucoma, including primary congenital glaucoma (PCG), exhibit defects in the conventional outflow pathway, resulting in reduced aqueous humor drainage and elevated intraocular pressure. Genome-wide association studies and large-scale genetic approaches have identified numerous glaucoma associated genes, among which SVEP1 encodes a large extracellular matrix protein expressed in the trabecular meshwork (TM) (Karpanen et al., 2017). It has been shown that neural crest-specific deletion of Angpt1 or Svep1, causes severe defects in the adjacent Schlemm’s canal, ocular hypertension and phenotypes resembling PCG of mice (Young et al., 2020). Furthermore, Thomson et al. combined conditional knockout genetics with scRNA-seq to dissect TM–SC signaling. Tissue-specific deletion of *Angpt1* or *Svep1* from TM resulted in primary congenital glaucoma (PCG)-like phenotypes with severe SC developmental defects (Thomson et al., 2021). Comparative single-cell transcriptomics of normal versus *Angpt1*-deficient eyes identified distinct TM and SC populations and revealed additional TM–SC signaling pathways. Functional validation demonstrated that recombinant ANGPT1 mimetic proteins promoted SC development, lowered IOP and protected RGCs in PCG models while also reducing IOP in healthy adult mice. This study represents a critical advance from descriptive atlases to mechanistic resolution, establishing ANGPT1-TEK signaling as a core regulatory axis for IOP homeostasis. However, whether similar mechanisms operate in adult-onset POAG remains to be determined. The Thomson study provides compelling evidence for a causative role of ANGPT1-TEK signaling in IOP regulation, supported by both genetic loss-of-function and pharmacological gain-of-function experiments. However, whether similar mechanisms operate in adult-onset POAG remains to be determined, as most POAG is not driven by developmental defects in the TM-SC junction. Additionally, the study was performed exclusively in mice; human validation is needed to confirm that ANGPT1 agonists would be effective in human disease.

### 2.3 Contractile dysfunction in TM

The sieve-like structure of the TM plays a critical role in regulating aqueous humor outflow resistance. Previous studies have reported that this structure possesses relaxation and contraction capabilities under mechanical stress, alongside an actin cytoskeleton architecture; however, the specific cell types and functional properties underlying TM structural dynamics remain poorly understood (Buffault et al., 2020). Jia et al. applied scRNA-seq to compare TM from spontaneous POAG and healthy non-human primates (NHPs), identifying 14 cell types and differential expression of contractility-related genes (*TPM1*, *ACTC1*, *TNNT1*) in POAG (Jia et al., 2023). Histological analysis revealed TM tissue densification and collapse, suggesting that contractile dysfunction contributes to increased outflow resistance. This study addressed a critical gap in disease-state single-cell analysis of the AHOP, providing evidence for a mechanistic link between cytoskeletal dynamics and drainage capacity. Greatbatch et al. performed high-throughput functional analysis by knocking out 62 genes at IOP-associated genome-wide association study (GWAS) loci in primary cultured human TM cells, followed by scRNA-seq and single-cell morphological analysis (Greatbatch et al., 2024). This approach prioritized *ANGPTL2*, *LMX1B*, *CAV1*, and *KREMEN1* as POAG candidate genes and revealed three genetic networks with cooperative functions in TM physiology. This study integrated CRISPR-based functional genomics with single-cell transcriptomics, systematically testing genetic perturbations to infer causality. The identification of *LMX1B* is particularly notable given its independent validation as a key transcription factor for TM mitochondrial function (Tolman et al., 2026).

Both studies suggest that contractile dysfunction and cytoskeletal remodeling are associated with POAG pathophysiology. However, the Jia study is correlative-it identifies differentially expressed genes but does not establish whether these changes are causal or compensatory. The Greatbatch study provides stronger causal inference through functional perturbation, but it was performed in cultured primary cells, which may not fully recapitulate the *in vivo* TM microenvironment. Together, these studies implicate cytoskeletal regulation as a potential therapeutic target, but further *in vivo* validation is required.

### 2.4 TM aging and mitochondrial dysfunction

The prevalence of glaucoma increases with advancing age. Some researchers have proposed that the number and density of trabecular meshwork cells decrease with senescence, resulting in compromised aqueous humor outflow tissue function (Croft et al., 2017). Other studies suggest that accumulation and alterations of the extracellular matrix during aging represent critical factors contributing to intraocular pressure fluctuations. ECM remodeling, involving changes in its composition and distribution, leads to reduced physiological filtration capacity of the trabecular meshwork (Liu et al., 2018). However, the underlying molecular mechanisms driving the age-dependent increase in glaucoma incidence remain incompletely understood. Wu et al. constructed a dynamic single-cell transcriptomic atlas of aged macaque TM, identifying 12 cell subtypes and establishing mitochondrial dysfunction as a hallmark of TM aging for the first time. Deep analysis of the most senescent cell cluster identified *APOE* as a key differentially expressed gene (Wu et al., 2024). *APOE* silencing enhanced cell migration, reduced apoptosis, and increased drainage capacity via PI3K–AKT pathway modulation and ECM component downregulation. This study introduces aging as a critical variable in AHOP single-cell analysis, consistent with clinical statistics showing increased POAG incidence in elderly patients. The convergence with Tolman et al. on mitochondrial dysfunction suggests that aging and genetic susceptibility may converge on common cellular stress pathways (Tolman et al., 2026). Tolman et al. performed multimodal single-cell analysis of mouse TM cells combining scRNA-seq with ATAC-seq. They identified three TM subtypes (TM1: ECM metabolism; TM2: secretory signaling; TM3: contractile/mitochondrial) and established *LMX1B* as a key transcription factor for mitochondrial function, with highest expression in TM3 cells. *Lmx1b* mutation caused TM3-specific mitochondrial pathway alterations, mitochondrial swelling, and reduced cristae area. Notably, vitamin B3 (nicotinamide) treatment prevented IOP elevation by enhancing mitochondrial function. This study provides a framework for understanding how distinct TM biological aspects are compartmentalized among specialized cell populations. The therapeutic demonstration that nicotinamide-a safe, inexpensive supplement-can prevent IOP elevation in genetic models opens direct translational possibilities.

The convergence of the Wu and Tolman studies on mitochondrial dysfunction suggests that aging and genetic susceptibility may converge on common cellular stress pathways. However, several caveats apply. The Wu study was performed in macaques, which are phylogenetically closer to humans than rodents but still not human. The Tolman study was performed in mice and focused on a specific genetic model (Lmx1b mutation); whether nicotinamide would be effective in sporadic, age-related POAG remains unknown. Furthermore, both studies identify associations between mitochondrial dysfunction and TM pathology, but the causal direction-whether mitochondrial dysfunction drives TM dysfunction or is a consequence of other cellular stressors-has not been fully established.

### 2.5 Spatial transcriptomics of the AHOP

Yuan et al. employed Stereo-seq nanoscale-resolution spatial transcriptomics for *in situ* analysis of the aqueous humor circulatory system. From two tissue chips, they segmented 60, 638 putative single cells, identifying 15 cell clusters including TM cells precisely localized to the iridocorneal angle (Yuan et al., 2025). This study addresses a key limitation of dissociation-based scRNA-seq: loss of spatial information. By preserving anatomical context, Yuan et al. confirmed the expected localization of TM cells and identified spatially restricted cell–cell interactions not inferable from dissociated cells. The relatively low number of genes detected per cell (445.3) reflects the current technical trade-off between spatial resolution and transcriptomic depth.

Spatial transcriptomics represents an important complementary approach to dissociated scRNA-seq. However, current spatial platforms face significant limitations, including lower gene detection sensitivity, higher cost and limited availability of fresh-frozen tissue. Moreover, spatial transcriptomics remains largely descriptive; it does not provide functional or causal information. The field would benefit from integration of spatial data with functional perturbations (e.g., CRISPR screens or pharmacological interventions) to move beyond correlation toward causation. Beyond the Yuan study, recent spatial transcriptomic investigations in retinal neurodegeneration have revealed regional vulnerability patterns and cell-cell interaction networks that cannot be resolved by dissociation-based methods, further emphasizing the value of spatial approaches.

Collectively, these studies demonstrate that TM cell heterogeneity far exceeds previous understanding, with functionally specialized subtypes regulating different aspects of drainage physiology. SC represents a unique vascular/lymphatic hybrid with distinct molecular properties. Mitochondrial dysfunction emerges as a common theme across aging, genetic and potentially acquired TM dysfunction. Cytoskeletal contractility and ECM remodeling are consistently associated with outflow resistance. ANGPT1-TEK and LMX1B represent druggable nodes for therapeutic intervention. However, the field is dominated by descriptive atlases and correlative studies; causal validation remains limited to a small number of investigations. The translation of these findings to human sporadic POAG-rather than monogenic or animal models-remains a major challenge.

## 3 Single-cell insights into POAG

POAG, the most common glaucoma subtype, is characterized by progressive RGC death and varying degrees of IOP elevation. While bulk transcriptomics has revealed differential expression profiles and implicated mitochondrial dysfunction, oxidative stress, neuroinflammation and metabolic disturbance in POAG, it masks cellular heterogeneity and precludes precise definition of causal roles for specific subpopulations. scRNA-seq has illuminated contributions of cholesterol metabolism, immune dysregulation and neuroinflammation to RGC degeneration, while also revealing important associations that require further functional validation.

### 3.1 Cholesterol homeostasis and RGC protection

Yang et al. investigated the functional consequences of *ABCA1*-associated POAG risk. POAG risk alleles near *ABCA1* reduced its expression; patients exhibited decreased ABCA1, reduced high-density lipoprotein (HDL) and elevated leukocyte cholesterol (Yang et al., 2023). *Abca1* knockout mice displayed retinal cholesterol accumulation and progressive age-dependent RGC loss. scRNA-seq revealed aberrant oxidative phosphorylation, mTORC1 activation and autophagy suppression. Atorvastatin treatment reduced retinal cholesterol, improved metabolism, and protected RGCs. This study exemplifies the translation from GWAS association to mechanistic understanding via scRNA-seq, although the mouse model represents complete Abca1 deficiency rather than the partial loss-of-function associated with human risk alleles. The convergence of cholesterol metabolism, mitochondrial function and autophagy aligns with emerging themes in neurodegeneration research. The demonstration that widely used, well-tolerated statins can prevent glaucoma-like changes in genetic models has direct clinical relevance. However, the study was performed in a complete knockout model, which may not accurately reflect the modest effects of human risk variants. Moreover, whether cholesterol accumulation is a primary driver of RGC degeneration or a secondary consequence of metabolic stress remains unclear. The association between ABCA1 variants and POAG is well-established, but the causal pathway from cholesterol dysregulation to RGC death requires further validation in human tissues.

### 3.2 Integrating genetic regulation and single-cell expression

Hamel et al. integrated GWAS, eQTL/sQTL colocalization, Mendelian randomization, and single-nucleus RNA sequencing (snRNA-seq) of glaucoma-relevant tissues to prioritize causal genes for 60% of POAG/IOP loci (Hamel et al., 2024). POAG/IOP colocalized genes were enriched in specific cell types across the outflow pathway, retina, optic nerve head, peripapillary sclera, and choroid. This study represents systematic integration of genetic and single-cell data, providing cell-type-specific mechanistic hypotheses for hundreds of genetic associations. Multi-tissue analysis revealed that POAG involves both IOP-dependent (outflow pathway) and IOP-independent (retina, optic nerve head) mechanisms, reconciling clinical observations of pressure-independent progression. Compared with Yang et al. (2023), which focused on a single gene, this study provides a systems-level perspective, though the reliance on statistical inference rather than experimental validation means many prioritized genes require functional confirmation.

Compared with the focused analysis of Yang et al. on a single gene, the Hamel study provides a systems-level perspective. However, the reliance on statistical inference (colocalization, Mendelian randomization) rather than experimental validation means many prioritized genes require functional confirmation. The study identifies associations, not causal mechanisms, and the statistical power of colocalization analyses depends on the quality of both GWAS and eQTL datasets. Furthermore, snRNA-seq of postmortem human tissues may not capture dynamic disease processes or acute responses to IOP elevation. Nonetheless, this study provides an invaluable resource for hypothesis generation and illustrates the power of integrating genetics with single-cell transcriptomics.

### 3.3 Circulating immune landscape in POAG

Tian et al. performed high-resolution scRNA-seq on approximately 1.4 million peripheral blood mononuclear cells (PBMCs) from 110 POAG patients and 110 matched controls (Tian et al., 2025). POAG was characterized by immune remodeling, including increased CD4+ T lymphocytes and myeloid cells, reduced terminally differentiated CD8+ GZMK+ T cells and natural killer (NK) cells, and a complex dual transcriptional landscape where pro-inflammatory and neuroprotective signaling pathways coexisted across multiple immune lineages. Cell-type-specific eQTL and summary-based Mendelian randomization (SMR) analyses revealed that POAG genetic risk variants may exert their effects through immune gene regulation in specific PBMC subpopulations. Functional validation in *Ifng*−/− and *Tnf*+/− mice demonstrated that genetic deficiency in these pathways exacerbated RGC loss and visual pathway defects. This is the largest single-cell immune analysis in glaucoma to date and the first to connect POAG genetic risk with immune cell-type-specific regulation. The discovery of systemic immune dysregulation challenges the traditional view of glaucoma as a purely ocular disease. However, several important caveats apply. First, the observed immune changes are associative; whether they are primary drivers of disease or secondary to neurodegeneration remains unclear. The mouse functional validation-showing that Ifng or Tnf deficiency exacerbates RGC loss-suggests a causal role for these cytokines, but it does not establish that the PBMC changes observed in patients are causative. Second, the dual pro-inflammatory and neuroprotective signature suggests that immune responses may be context-dependent, complicating therapeutic targeting. Third, PBMC composition may be influenced by medications, comorbidities, and other factors not fully controlled for in the study design. Nonetheless, the study provides compelling evidence that systemic immunity warrants further investigation as a contributor to POAG pathogenesis.

### 3.4 ATOH7-mediated neuroprotection

Yin et al. integrated human retinal scRNA-seq data with GeneCards and performed scRNA-seq on *Atoh7* knockout mice, identifying *ATOH7* as a key gene (Yin et al., 2023). Pseudotemporal analysis revealed altered cell differentiation in *Atoh7*-deficient retina. *In vitro* experiments confirmed that ATOH7 overexpression inhibited Notch signaling, promoted photoreceptor differentiation, and suppressed apoptosis. This study highlights the potential of scRNA-seq data mining for target discovery, connecting developmental and degenerative mechanisms. While this study identifies an interesting association between ATOH7 and photoreceptor survival, its direct relevance to glaucomatous RGC degeneration is limited, as the study focused primarily on photoreceptors rather than RGCs. Furthermore, the *in vitro* overexpression experiments do not recapitulate the complex cellular environment of the glaucomatous retina. The Notch pathway has been implicated in glaucoma by other studies (Teotia et al., 2020), suggesting that developmental signaling pathways may be reactivated in disease, but the causal role of ATOH7 specifically in RGC neuroprotection remains to be established in relevant glaucoma models.

### 3.5 RGC subtype susceptibility and resilience

While the review has mentioned RGC heterogeneity, recent studies have begun to dissect the differential vulnerability of RGC subtypes to glaucomatous injury-a topic with profound implications for neuroprotective strategies. Over 45 RGC subtypes have been identified in mice, each with distinct molecular signatures, connectivity patterns, and susceptibility to injury (Sanes & Masland, 2015). Nimkar et al. employed molecular and spatial analysis of retinal flatmounts to identify perivascular RGCs that are resilient to glaucoma (Nimkar et al., 2025). These resilient RGCs were characterized by high expression of Oprm1 (opioid receptor mu 1) and other genes associated with metabolic flexibility and calcium buffering. Conversely, RGC subtypes with high metabolic demands and low antioxidant capacity appear to be preferentially vulnerable. Qian et al. constructed an intercellular communication atlas and identified OPRM1 as a neuroprotective factor for RGCs (Qian et al., 2024). This study demonstrated that Müller glia-derived signals may influence RGC survival through OPRM1-mediated pathways. These findings suggest that differential vulnerability is not purely cell-intrinsic but is modulated by local microenvironment and cell–cell communication.

The identification of resilient RGC subtypes raises the possibility of subtype-targeted neuroprotection-for example, by upregulating resilience-associated genes in vulnerable populations. However, several challenges remain. First, the relationship between RGC subtype vulnerability in mice and in humans is not fully established; humans have additional RGC subtypes and a fovea, which may alter susceptibility patterns. Second, the molecular correlates of resilience are largely associative; it is unclear whether Oprm1 expression causally confers protection or is simply a marker of a resilient state. Third, translating subtype-specific findings to therapy will require delivery systems that can target specific RGC populations, which is technically challenging. Nonetheless, this area represents a promising frontier for neuroprotective strategy development.

### 3.6 Optic nerve head and lamina cribrosa remodeling

The optic nerve head (ONH) and lamina cribrosa (LC) are increasingly recognized as critical sites of glaucomatous injury, where RGC axons are mechanically stressed and glial remodeling occurs. However, single-cell studies of the ONH and LC remain relatively sparse compared to those of the retina and TM. Existing studies have identified ONH astrocytes as key mediators of mechanical stress responses, with evidence for astrocyte activation, ECM remodeling, and glial scar formation in glaucoma (reviewed in several bulk and single-cell studies). The lamina cribrosa, composed of astrocyte-like cells and ECM, undergoes thickening and reorganization in glaucoma, contributing to axonal compression and impaired axoplasmic flow.

Recent single-cell and spatial transcriptomic studies have begun to characterize the cellular composition of the ONH, revealing heterogeneity among ONH astrocytes and their differential responses to IOP elevation. These studies suggest that ONH glial remodeling involves both reactive gliosis and adaptive responses that may initially protect axons but ultimately contribute to pathology. The relative contributions of ONH astrocytes versus LC cells to axonal injury remain incompletely defined. The ONH and LC represent a major gap in the single-cell literature on glaucoma. Most studies focus on either the anterior segment (TM/SC) or the retina (RGCs and Müller glia), leaving the intermediate site of axonal injury relatively unexplored at single-cell resolution. Technical challenges—including the small size of the ONH, its complex three-dimensional structure, and the difficulty of obtaining high-quality human ONH tissue—have limited progress. The field would benefit from dedicated single-cell and spatial transcriptomic studies of the ONH and LC across disease stages, as well as integration with biomechanical modeling to link physical stress to cellular responses.

Collectively, the studies discussed in this section reveal that: (1) cholesterol metabolism, particularly ABCA1-mediated HDL biogenesis, is associated with RGC survival; (2) POAG involves both local ocular and systemic immune dysregulation; (3) genetic risk variants may exert cell-type-specific effects across multiple tissues; (4) NF-κB, Hippo, mTOR, and Notch pathways represent convergent signaling nodes of interest; and (5) repurposable drugs (statins, nicotinamide) show promise in preclinical models. However, key controversies remain: whether immune changes are primary or secondary, the relative contributions of IOP-dependent versus IOP-independent mechanisms, whether RGC subtype vulnerability in mice translates to humans, and the challenge of translating animal findings to human disease. The ONH and LC represent critical but underexplored sites of pathology.

## 4 Discussion

### 4.1 Convergent pathogenic mechanisms and open controversies

Systematic comparative analysis of recent scRNA-seq studies across glaucoma models and subtypes reveals several convergent pathogenic themes, alongside important controversies that remain unresolved.

Mitochondrial dysfunction represents the most consistently identified mechanism, implicated in TM aging (Wu et al., 2024), genetic TM disease (Tolman et al., 2026), RGC cholesterol metabolism (Yang et al., 2023), high-IOP-induced metabolic stress (Xiao et al., 2025) and IR injury (Yang et al., 2023). The demonstration that nicotinamide (vitamin B3) can prevent IOP elevation by enhancing mitochondrial function in genetic models (Tolman et al., 2026), and that empagliflozin protects RGCs by restoring mitochondrial fusion (Yang et al., 2023), positions metabolic resilience as a promising therapeutic strategy across glaucoma subtypes. However, these findings are predominantly from animal models and require validation in human disease. Moreover, mitochondrial dysfunction may be a consequence rather than a cause of cellular stress in many contexts; the causal direction remains to be definitively established.

Neuroinflammation-particularly microglial activation-emerges as a common feature in POAG (Tian et al., 2025), NTG (Liu et al., 2025; Qiu et al., 2025), high-IOP models (Kumar et al., 2025), and even RGC transplantation (Kriukov et al., 2025). The identification of specific inflammatory nodes-including the NLRP3 inflammasome (Yang et al., 2023; Qiu et al., 2025), TNF-α (Liu et al., 2025), and NF-κB/Hippo pathways—provides multiple targets for anti-inflammatory intervention. However, the finding that some immune responses may be neuroprotective (Tian et al., 2025) cautions against indiscriminate immunosuppression. A critical unresolved question is whether neuroinflammation is a primary driver of neurodegeneration or a secondary response to injury-a distinction with major therapeutic implications.

Cell-cell communication remodeling has been identified across multiple studies, including altered Müller-RGC interactions in high-IOP models (Xiao et al., 2025), enhanced astrocyte/Müller-RGC communication in ONC (Qian et al., 2024) and disrupted neurexin/VEGF signaling in glaucoma (Xiao et al., 2025). These findings suggest that glaucoma involves not merely cell death but progressive remodeling of retinal circuits, with implications for both pathogenesis and therapeutic targeting. However, the functional significance of most identified ligand–receptor interactions has not been validated, and the directionality of communication changes (protective versus harmful) remains to be determined.

To better visualize the experimental heterogeneity and unresolved contradictions across studies, we summarize key studies in **Table 1**, highlighting discrepancies in species, platforms, and conflicting interpretations (e.g., pro-inflammatory vs. protective immunity, systemic vs. local mechanisms, and the translational gap between animal models and human disease).

**Table 1:** Comparative summary of key single-cell studies in AHOP and POAG.

Research area	Study (first author, year)	Species/sample	Cells/sample size	Platform	Main conclusions
AHOP - Cross-Species Atlas	Patel et al. (2020)	Human donor eyes	8, 758 cells	scRNA-seq (10x)	SC cells have hybrid vascular/lymphatic signature; TM shows substantial heterogeneity
	van Zyl et al. (2020)	Human, monkey, pig, mouse	~24, 000 cells	scRNA-seq	Identified 19 conserved cell types; many human orthologs exist but with expression differences
AHOP - TM–SC Signaling	Thomson et al. (2021)	Mouse (conditional KO)	Not specified	scRNA-seq + KO genetics	ANGPT1–TEK axis is core regulator of IOP; recombinant ANGPT1 lowers IOP and protects RGCs in PCG models
AHOP - Contractile Dysfunction	Jia et al. (2023)	Non-human primate (spontaneous POAG)	14 cell types identified	scRNA-seq	Contractile genes (*TPM1, ACTC1, TNNT1*) dysregulated in POAG; TM tissue densification and collapse
	Greatbatch et al. (2024)	Primary human TM cells (CRISPR)	62 genes knocked out	scRNA-seq + CRISPR	Prioritized *ANGPTL2, LMX1B, CAV1, KREMEN1* as causal POAG genes; identified 3 functional networks
AHOP - Aging & Mitochondria	Wu et al. (2024)	Aged macaque TM	12 subtypes	scRNA-seq	Mitochondrial dysfunction is hallmark of TM aging; *APOE* silencing improves drainage via PI3K–AKT
	Tolman et al. (2026)	Mouse TM	3 subtypes (TM1–TM3)	scRNA-seq + ATAC-seq	LMX1B controls mitochondrial function in TM3; nicotinamide prevents IOP elevation in genetic models
AHOP - Spatial Transcriptomics	Yuan et al. (2025)	Human AHOP tissue	60, 638 putative single cells	Stereo-seq (spatial)	Confirmed TM localization; identified spatially restricted cell–cell interactions
POAG - Cholesterol Metabolism	Yang et al. (2023)	Mouse (*Abca1* KO) + patient data	Not specified	scRNA-seq	ABCA1 deficiency causes cholesterol accumulation, mTORC1 activation, autophagy suppression; atorvastatin protects RGCs
POAG - Genetic Integration	Hamel et al. (2024)	Human (multiple tissues)	GWAS + snRNA-seq	snRNA-seq + eQTL/MR	Prioritized causal genes for 60% of POAG/IOP loci; both IOP-dependent and -independent mechanisms
POAG - Systemic Immunity	Tian et al. (2025)	Human PBMCs (110 POAG vs. 110 controls)	~1.4 million cells	scRNA-seq (high-throughput)	Systemic immune remodeling in POAG; pro-inflammatory and neuroprotective signals coexist; *Ifng/Tnf* deficiency exacerbates RGC loss
POAG - ATOH7 Neuroprotection	Yin et al. (2023)	Human scRNA-seq data + *Atoh7* KO mice	Not specified	scRNA-seq + *in vitro*	ATOH7 inhibits Notch, promotes differentiation, suppresses apoptosis

Beyond the questions above, significant disagreements exist in the literature. Some studies emphasize systemic immune dysregulation as a driver of POAG (Tian et al., 2025), while others focus on local ocular inflammation (Liu et al., 2025; Qiu et al., 2025). The relative importance of IOP-dependent versus IOP-independent mechanisms remains debated, with some studies suggesting that pressure-independent pathways dominate in certain patient subgroups (Hamel et al., 2024). Whether RGC subtype vulnerability patterns in mice translate to humans remains unclear, particularly given the presence of foveal RGCs in humans that have no rodent counterpart. The translational gap between animal models and human disease is a recurring theme-van Zyl et al. identified significant species differences in AHOP cell types, and Budoff & Poleg-Polsky found limited correlation between mouse and primate retinal gene programs (Budoff and Poleg-Polsky, 2025). These controversies highlight the need for more human tissue studies, longitudinal analyses, and functional validation of single-cell findings.

### 4.2 Methodological considerations and technical limitations

Several methodological issues warrant critical evaluation.

Species translation remains a fundamental challenge: van Zyl et al. identified significant differences between human and animal model AHOP cell types (van Zyl et al., 2020), while Budoff and Poleg-Polsky found limited correlation between mouse area of temporal retina and primate macular gene programs (Budoff and Poleg-Polsky, 2025). Although animal models are essential for mechanistic and therapeutic studies, single-cell analysis of clinical specimens is ideally necessary to establish translational confidence. The field should prioritize studies using human postmortem or surgical tissues, complemented by animal models for functional validation.

Loss of spatial information is a persistent concern in scRNA-seq studies. Yuan et al. demonstrated that spatial transcriptomics can identify cell types and locations not inferable from dissociated cells (Yuan et al., 2025), while other studies have shown that cultured primary cells differ significantly from tissue-resident cells. Future investigations should increasingly integrate spatial transcriptomics to validate and extend dissociation-based findings. However, spatial approaches face their own limitations, including lower gene detection sensitivity and the need for fresh-frozen tissue. Regarding cell-type resolution versus depth, high-resolution studies identifying 45+ RGC subtypes provide exquisite detail but may lack statistical power to detect subtle expression changes (Nimkar et al., 2025). Conversely, studies aggregating cells into broad categories may miss subtype-specific effects. Optimal study designs should balance these considerations based on specific research questions.

Dissociation bias preferentially loses fragile cell types (e.g., RGCs, TM cells), potentially skewing subtype composition and susceptibility analyses. Many studies report low recovery of RGCs, which may affect the identification of vulnerable versus resilient subtypes. Optimized dissociation protocols and the use of single-nucleus RNA-seq (snRNA-seq) for fragile or frozen tissues can mitigate but not fully eliminate this bias.

Transcriptomic depth varies substantially across platforms. High-throughput scRNA-seq platforms (e.g., 10x Genomics) typically detect 1, 000–3, 000 genes per cell, while low-throughput full-length approaches (e.g., Smart-seq2) detect more genes but at lower throughput. Spatial approaches face even lower per-cell gene counts (~445 genes in the Yuan study), highlighting a persistent resolution-sensitivity trade-off. The choice of platform significantly influences the types of biological questions that can be addressed.

Batch effects from variable sample processing can confound disease–control comparisons. Most glaucoma studies do not explicitly report batch-correction strategies, and it is often unclear whether observed differences represent disease biology or technical artifacts. Transparent reporting of sample processing, sequencing batches, and correction methods is essential.

Analytical variability arising from divergent clustering algorithms, resolution parameters, and pseudotime methods—can yield inconsistent subtype definitions across studies, complicating meta-analyses. The field would benefit from standardized pipelines and benchmarking studies to improve cross-study comparability.

These technical limitations demand cautious interpretation of single-cell findings. Many reported cell-type-specific markers and disease-associated changes may reflect technical artifacts rather than biological reality. Orthogonal validation via spatial transcriptomics, histological staining, functional assays, or CRISPR perturbation should be performed to confirm key findings. The field should adopt transparent reporting standards, including detailed descriptions of dissociation, library preparation, sequencing depth, batch correction, and clustering parameters.

### 4.3 Spatial multi-omics and emerging technologies

Spatial transcriptomics has expanded substantially beyond the Yuan et al. study. Recent advances in spatial platforms-including Visium, MERFISH, and Stereo-seq-enable transcriptomic profiling with preserved tissue architecture at near-single-cell resolution. In the context of glaucoma, spatial approaches have been applied to both the anterior segment (AHOP) and posterior segment (retina, ONH), revealing region-specific gene expression patterns and cell–cell interactions that cannot be resolved by dissociation-based methods.

Key advantages of spatial transcriptomics include: (1) preservation of anatomical context, allowing precise localization of cell types to specific tissue structures (e.g., TM to the iridocorneal angle, RGC subtypes to specific retinal layers); (2) identification of spatially restricted cell–cell interactions; (3) correlation of gene expression with tissue histology and pathology; and (4) the potential to integrate with spatial proteomics and metabolomics for multi-modal characterization.

However, current spatial technologies face significant limitations: (1) lower gene detection sensitivity compared to dissociated scRNA-seq, limiting analysis of low-abundance transcripts; (2) limited ability to detect splice variants or allele-specific expression; (3) higher cost and lower throughput; (4) dependence on fresh-frozen or fixed tissue, limiting compatibility with archival samples; and (5) computational challenges in integrating spatial data with dissociated single-cell data.

The combination of spatial transcriptomics with functional perturbations-for example, via spatially targeted CRISPR screens or pharmacological interventions-represents an exciting frontier. Integration of spatial data with single-cell multi-omics could provide a comprehensive view of glaucoma pathophysiology *in situ*. The field should also work toward developing standardized spatial atlases of healthy and glaucomatous human eyes, analogous to the Human Cell Atlas, to facilitate data sharing and meta-analysis.

### 4.4 Multi-omics integration and epigenomic profiling

While scRNA-seq provides a high-resolution view of the transcriptome, it does not directly inform about chromatin accessibility, DNA methylation, or protein expression. Multi-omic approaches that integrate these layers of regulation are beginning to be applied in glaucoma research. The Tolman et al. study exemplifies the power of joint transcriptome-epigenome profiling by combining scRNA-seq with ATAC-seq (Assay for Transposase-Accessible Chromatin using sequencing) in mouse TM cells (Tolman et al., 2026). This approach identified LMX1B as a key transcription factor regulating mitochondrial function in TM3 cells, demonstrating how chromatin accessibility information can prioritize transcription factors and regulatory networks from transcriptomic data.

Emerging multi-omic platforms include: (1) 10x Multiome (scRNA-seq+scATAC-seq from the same single cell), allowing direct correlation of gene expression with chromatin accessibility; (2) CITE-seq (scRNA-seq+cell surface protein quantification), linking transcriptome to protein expression; (3) scRNA-seq+DNA methylation (e.g., scBS-seq), revealing epigenetic regulation; and (4) spatial multi-omics, combining spatial transcriptomics with spatial proteomics or metabolomics. Multi-omics integration is essential for distinguishing causal versus correlative changes in gene expression. For example, if a gene is upregulated in disease but its promoter is closed (inaccessible chromatin), the upregulation is likely secondary to other factors. Conversely, if a transcription factor is differentially expressed and its target sites show increased accessibility, this suggests a causal regulatory relationship. However, multi-omic approaches face significant challenges: higher cost, increased data complexity, computational demands for data integration and limited availability of tissue samples. The field should prioritize multi-omic studies in well-characterized human tissues to maximize translational relevance. Furthermore, functional validation (e.g., CRISPR perturbations, pharmacological interventions) remains essential to confirm causal relationships inferred from multi-omic data.

### 4.5 Future directions and clinical translation

Despite significant advances, critical gaps persist. Longitudinal single-cell analyses are largely absent; most studies capture single time points, limiting understanding of disease progression dynamics. Human disease tissue analysis remains underrepresented, with the vast majority of studies employing animal models. Multi-omics integration, while exemplified by Tolman et al. has not yet become standard practice, limiting mechanistic depth (Tolman et al., 2026). The clinical translation pathway from single-cell discovery to therapeutic validation remains poorly defined, with few studies advancing from target identification to preclinical efficacy testing. Several emerging directions promise to advance the field. Spatial multi-omics combining transcriptomics, proteomics, and epigenomics with preserved tissue architecture will provide unprecedented *in situ* resolution of glaucoma pathophysiology. Large-scale single-cell CRISPR screens will enable systematic functional dissection of genetic risk variants (Greatbatch et al., 2024). Machine learning integration of single-cell data with clinical parameters may enable precise classification of glaucoma subtypes and prediction of treatment responses. Liquid biopsy approaches analyzing circulating cell-free RNA or extracellular vesicles could provide minimally accessible biomarkers reflecting intraocular single-cell states.

## 5 Conclusion

Single-cell transcriptomics has fundamentally transformed glaucoma research by revealing cellular heterogeneity, identifying shared pathogenic mechanisms and discovering therapeutic targets inaccessible to bulk analysis. The convergence across diverse glaucoma subtypes and models on mitochondrial dysfunction, neuroinflammation and altered cell–cell communication suggests shared terminal pathways amenable to unified therapeutic strategies. As the field advances toward spatial multi-omics, longitudinal analysis and clinical translation, single-cell technologies promise to enable precision medicine approaches that match specific glaucoma pathophysiologies to optimally targeted interventions. Realizing this potential will require sustained investment in human tissue analysis, methodological innovation, and rigorous preclinical validation of single-cell-derived therapeutic hypotheses.
